# The role of abnormalities of lipoproteins and HDL functionality in small fibre dysfunction in people with severe obesity

**DOI:** 10.1038/s41598-021-90346-9

**Published:** 2021-06-15

**Authors:** Shazli Azmi, Maryam Ferdousi, Yifen Liu, Safwaan Adam, Tarza Siahmansur, Georgios Ponirakis, Andrew Marshall, Ioannis N. Petropoulos, Jan Hoong Ho, Akheel A. Syed, John M. Gibson, Basil J. Ammori, Paul N. Durrington, Rayaz A. Malik, Handrean Soran

**Affiliations:** 1 Division of Cardiovascular Sciences, Cardiac Centre, Faculty of Biology, Medicine and Health, The University of Manchester, Manchester, UK; 2 Cardiovascular Trials Unit, The Old St Mary’s Hospital, Central Manchester University Hospitals, Manchester, M13 9WL UK; 3 The Christie NHS Foundation Trust, Manchester, UK; 4 Weill Cornell Medicine-Qatar, Doha, Qatar; 5 Institute of Life Course and Medical Sciences, University of Liverpool, Liverpool, UK; 6 Department of Diabetes and Endocrinology, Salford Royal Trust NHS Foundation Trust, Salford, UK; 7 Department Surgery, Salford Royal Trust NHS Foundation Trust, Salford, UK; 8 Diabetes, Endocrine and Metabolism Centre, Manchester University NHS Foundation Trust, Manchester, UK

**Keywords:** Obesity, Weight management

## Abstract

Obesity and associated dyslipidemia may contribute to increased cardiovascular disease. Obesity has also been associated with neuropathy. We have investigated presence of peripheral nerve damage in patients with severe obesity without type 2 diabetes and the status of metabolic syndrome and lipoprotein abnormalities. 47participants with severe obesity and 30 age-matched healthy controls underwent detailed phenotyping of neuropathy and an assessment of lipoproteins and HDL-functionality. Participants with severe obesity had a higher neuropathy symptom profile, lower sural and peroneal nerve amplitudes, abnormal thermal thresholds, heart rate variability with deep breathing and corneal nerve parameters compared to healthy controls. Circulating apolipoprotein A1 (*P* = 0.009), HDL cholesterol (HDL-C) (*P* < 0.0001), cholesterol efflux (*P* = 0.002) and paroxonase-1 (PON-1) activity (*P* < 0.0001) were lower, and serum amyloid A (SAA) (*P* < 0.0001) was higher in participants with obesity compared to controls. Obese participants with small nerve fibre damage had higher serum triglycerides (*P* = 0.02), lower PON-1 activity (*P* = 0.002) and higher prevalence of metabolic syndrome (58% vs. 23%, *P* = 0.02) compared to those without. However, HDL-C (*P* = 0.8), cholesterol efflux (*P* = 0.08), apoA1 (*P* = 0.8) and SAA (*P* = 0.8) did not differ significantly between obese participants with and without small nerve fibre damage**.** Small nerve fibre damage occurs in people with severe obesity. Patients with obesity have deranged lipoproteins and compromised HDL functionality compared to controls. Obese patients with evidence of small nerve fibre damage, compared to those without, had significantly higher serum triglycerides, lower PON-1 activity and a higher prevalence of metabolic syndrome.

## Introduction

Obesity is a worldwide epidemic conferring a major public health challenge, placing increased economic burden on health care systems, and is the fifth leading global cause of death from cardiovascular disease and cancer^1^.

Type 2 diabetes (T2DM) is the commonest cause of peripheral neuropathy (PN), but impaired glucose tolerance (IGT), hypertriglyceridemia and increased waist circumference are also associated with PN^2–5^. The Monica/Kora Augsburg study showed an increased prevalence of painful neuropathy in people with IGT, which was associated independently with weight and waist circumference^5^. The Cooperative Health Research in the Region of Augsburg (KORA) F4 study also showed that the prevalence of PN in elderly people with IGT and T2D was comparable^6^ and that abdominal obesity was associated with the development of diabetic polyneuropathy (DPN)^7^. The mechanisms that drive obesity-related neuropathy have received limited attention.

Small fibre neuropathy and cardiac autonomic neuropathy occur in obese individuals with and without T2DM^8–11^. Hypertriglyceridemia and obesity have been associated with reduced intraepidermal nerve fibres and elevated HbA_1c_ has been related to reduced nerve conduction velocity^12^. We have previously shown small fibre pathology in people with IGT^13^, especially those who progress to diabetes^14^. Intraepidermal nerve fibre density increases after diet and exercise in people with IGT^15^, particularly those with the metabolic syndrome^16^ and small nerve fibres regenerate after bariatric surgery^17^.

Dyslipidaemia has previously been implicated in the pathogenesis of diabetic neuropathy^18^. Serum paraoxonase-1 (PON-1), an antioxidant and antiatherogenic component of HDL reduces the susceptibility of LDL to lipid peroxidation^19^ and is lower in people with diabetes and microvascular complications^20^. Short-term improvements in serum triglycerides improve diabetic neuropathy^21^. High-fat fed mice with peripheral nerve and dorsal root ganglion overexpression of 12/15-lipoxygenase develop small nerve fibre damage^22^. A loss of HDL’s antioxidant function and systemic and adipose tissue inflammation may also contribute to obesity-mediated neuropathy^23,24^.

We hypothesised that neuropathy and small nerve fibre damage in particular, is associated with abnormalities in circulating lipoproteins, HDL-functionality and metabolic syndrome in people with severe obesity.

## Materials and methods

Participants with obesity were recruited from a regional tier 3 specialist weight management service at Salford Royal Hospital and were not previously diagnosed with T2D or prediabetes, confirmed by an HbA1c < 42 mmol/mol (6.0%). The control group were healthy volunteers recruited from Manchester University Foundation Trust, Salford Royal Hospital and the University of Manchester. Exclusion criteria were history of cancer, previous chemotherapy or radiotherapy, diabetes mellitus, prediabetes, anaemia, hereditary neuropathies, inborn errors of metabolism, untreated vitamin and mineral deficiencies, low vitamin B_12_ or folate levels, history of corneal trauma or surgery or a history of ocular or systemic disease that may affect the cornea. This study was approved by the Central Manchester Research and Ethics Committee. This research adhered to the tenant of declaration of Helsinki and was carried out in accordance with the relevant guidelines and regulations. All subjects provided written informed consent prior to the participation.

## Demographics and assessment of neuropathy

All study participants underwent assessment of body mass index (BMI (kg/m^2^)) , blood pressure (Dinamap pro 100v2, GE Medical Systems, Freiburg, Germany) and neuropathy symptoms using the Neuropathy symptom profile (NSP). Neurological deficits were evaluated using the modified neuropathy disability score (NDS), which is comprised of an assessment of vibration perception, pin-prick, temperature sensation and presence or absence of ankle reflexes^25^. A Neurothesiometer (Horwell, Scientific Laboratory Supplies, Wilfrod, Nottingham, UK) was used for the assessment of vibration perception threshold (VPT) and TSA-II NeuroSensory Analyser (Medoc Ltd., Ramat-Yishai, Israel) for the assessment of cold (CPT) and warm (WPT) perception thresholds^14^.

Electro-diagnostic studies were undertaken using a Dantec “Keypoint” system (Dantec Dynamics Ltd, Bristol, UK) equipped with a DISA temperature regulator to keep limb temperature constantly between 32 and 35 °C. Sural sensory nerve amplitude, conduction velocity and latency and peroneal motor nerve amplitude, conduction velocity and latency were assessed by a consultant neurophysiologist using a Dantec “Keypoint” system (Dantec Dynamics Ltd, Bristol, UK)^14^.

Heart rate variability with deep breathing (HRV-DB) was assessed with an ANX 3.0 autonomic nervous system monitoring device (ANSAR Medical Technologies Inc., Philadelphia, PA, USA).

Patients underwent examination with a corneal confocal microscope (CCM; Heidelberg Retinal Tomograph III Rostock Cornea Module, Heidelberg Engineering GmbH, Heidelberg, Germany) as per our previously established protocol^26^. Six non-overlapping images/patient (3 per eye) were selected from the central cornea following an established protocol^27^. Manual corneal nerve quantification was undertaken using CCMetrics (University of Manchester, UK) in a masked fashion. Corneal nerve fiber density (CNFD)—the total number of major nerves/mm^2^ of corneal tissue, corneal nerve branch density (CNBD)—the number of branches emanating from the major nerve trunks/mm^2^ of corneal tissue and corneal nerve fiber length (CNFL)—the total length of all nerve fibers and branches (mm/mm^2^) within the area of corneal tissue were assessed. Subjects with obesity were divided into those with and without small fibre damage based on a CNFL < 2SD of the control mean.

### Blood sampling

Blood samples were collected after a 10 h overnight fast, serum and EDTA-plasma were isolated by centrifugation at 2000 g for 15 min at 4° C within 2 h of collection and stored at -70^0^C.

### Lipid Profile

Total cholesterol was measured using the cholesterol oxidase phenol 4-aminoantipyrine peroxidase method, serum triglycerides by the glycerol phosphate oxidase phenol 4-aminoantipyrine peroxidase method, and apolipoprotein A1 (apoA1) was assayed using immunoturbidimetric assays. HDL-C was assayed using the direct clearance method. All these tests were performed on a Randox daytona analyzer (Randox Laboratories Ltd, Crumlin, County Antrim, UK). The laboratory participates in RIQAS (Randox International Quality Assessment Scheme; Randox Laboratories, Dublin, Ireland), which is CRC calibrated. LDL was estimated using the Friedewald formula.

### Paraoxonase-1 (PON-1) activity

Serum PON-1 activity was determined by a semi-automated micro-titer plate method using paraoxon (O,O-Diethyl O-4-nitrophenyl phosphate^28^. Plates were read at 405 nm using a multiskan multisoft plate reader (Labsystems, Hampshire, UK). Intra-assay and inter-assay CVs were 3% and 3.5%, respectively.

### Serum amyloid A (SAA)

SAA was measured using the human SAA solid-phase sandwich ELISA (ThermoFisher Scientific, Loughborough, UK). Intra-assay and inter-assay CVs were 6.1% and 7.4%, respectively.

### Capacity of HDL to promote cholesterol efflux in vitro

Cholesterol efflux of HDL was determined in an assay that has been previously validated^29^. ApoB depleted serum was prepared after removal of apolipoprotein B (apoB) containing lipoproteins with polyethylene glycol (MW 8000; Sigma),diluted to 2.8%^30^ and J774A.1 cells were incubated with radiolabelled cholesterol and incubated with apoB-depleted serum for 4 h. After incubation, the cell media was collected, and cells were washed with PBS and dissolved in 0.5 ml 0.2 N NaOH to determine radioactivity. Cellular cholesterol efflux was expressed as the percentage of radioactivity in the medium from the radioactivity in the cells and medium. Cholesterol efflux was linear over 4 h and was calculated using the following formula:

$$Cholesterol\, efflux \left( \% \right) = \frac{radioactivity\, in\, medium}{{radioactivity\, in\, cell + radioactivity\, in\, medium}} \times 100$$

To calculate cholesterol efflux at any time point, we subtracted efflux to serum free media (control) from that of apolipoprotein B depleted serum. Intra-assay and inter-assay coefficient of variance (CV) were 3.9% and 7.3%, respectively.

### Statistical analysis

Analysis was carried out on SPSS for Mac (Version 19.0, IBM Corporation, New York, USA). All data are expressed as mean ± standard deviation (SD). The data were assessed for normality and appropriate statistical analyses conducted. To assess within and between group differences we used one-way analysis of variance (ANOVA) or a non-parametric counterpart (Kruskal–Wallis). A significant *p* value was considered to be < 0.05.

## Results

### Clinical variables

We studied 47 participants with severe obesity compared to 30 age-matched healthy controls (P = 0.5) (Table 1). The obese group had a significantly higher weight (P < 0.0001), waist circumference (P < 0.0001) and BMI (P < 0.0001), but no statistically significant difference in HbA_1c_, blood pressure or eGFR compared to controls **(**Table 1). Nineteen (40%) of the participants with obesity fulfilled the criteria for metabolic syndrome^31^.

**Table 1:** Clinical, metabolic and neuropathy measures in controls and participants with obesity.

Parameters	Control (n = 30)	Obese (n = 47)	P
*Demographics*			
Age (years)	45.8 ± 8.9	47.1 ± 9.4	0.6
Sex (Female/Male)	17/13	30/17	0.1
Ethnicity (Caucasian/Asian)	25/5	43/4	0.1
Smoking (no. per day)	0.6 ± 2.3	1.3 ± 4.3	0.8
Alcohol (units per week)	2.8 ± 6.3	1.5 ± 3.2	0.2
Height (cm)	167.0 ± 10.4	166.9 ± 12.3	0.8
Waist circumference (cm)	90.5 ± 13.6	133.2 ± 14.9	< 0.0001
BMI (kg/m^2^)	26.4 ± 4.2	49.3 ± 8.3	< 0.0001
HbA1c (mmol/mol)	37.4 ± 3.9	37.9 ± 5.2	0.4
Systolic BP (mmHg)	127.2 ± 20.1	129.8 ± 19.7	0.5
Diastolic BP (mmHg)	74.3 ± 9.3	72.6 ± 10.5	0.8
eGFR (ml/min/l)	84.0 ± 10.9	82.0 ± 27.4	0.6
Number (%) on statin therapy	0 (0)	11^23^	< 0.0001
Number (%) with metabolic syndrome	0(0)	19^40^	< 0.0001
Total Cholesterol (mmol/l)	5.1 ± 0.9	4.7 ± 1.1	0.002
Serum triglycerides (mmol/l)	1.4 ± 0.6	1.7 ± 1.1	0.4
HDL-C (mmol/l)	1.5 ± 0.4	1.0 ± 0.3	< 0.0001
LDL-C (mmol/l)	2.9 ± 0.9	2.9 ± 0.8	0.6
Non-HDL-C (mmol/L)	3.6 ± 1.1	3.5 ± 1.4	0.6
*Neuropathy assessments*			
Neuropathy Symptom Profile	0.3 ± 1.0	3.9 ± 4.8	< 0.0001
Neuropathy Disability Score	0.4 ± 0.9	1.2 ± 2.0	0.08
Vibration Perception Threshold (volts)	5.4 ± 3.4	10.8 ± 7.1	< 0.0001
Sural Amplitude (μV)	21.8 ± 10.0	11.3 ± 8.8	< 0.0001
Sural Velocity (m/s)	51.6 ± 4.7	49.1 ± 8.6	0.1
Peroneal Amplitude (mV)	5.7 ± 2.1	3.8 ± 2.1	0.006
Peroneal Velocity (m/s)	49.2 ± 3.9	46.6 ± 5.0	0.07
Cold Perception Threshold (^O^C)	28.3 ± 2.7	25.4 ± 5.4	0.003
Warm Perception Threshold (^O^C)	37.1 ± 2.4	40.6 ± 3.3	< 0.0001
HRV-DB (beats per min)	33.8 ± 13.0	19.4 ± 11.0	< 0.0001
CNFD (no/mm^2^)	39.4 ± 6.2	26.7 ± 4.8	< 0.0001
CNBD (no/mm^2^)	110.4 ± 35.1	57.5 ± 25.4	< 0.0001
CNFL (mm/mm^2^)	29.2 ± 4.1	18.4 ± 3.9	< 0.0001

Results reported as mean ± standard deviation; BMI (body mass index); BP ( blood pressure); eGFR (estimated glomerular filtration rate); HDL-C (high density lipoprotein cholesterol); LDL-C (low density lipoprotein cholesterol); HRV-DB (Heart rate variability-deep breathing; CNFD (corneal nerve fibre density); CNBD (corneal nerve branch density); CNFL (corneal nerve fibre length).

### Neuropathy assessment in obese compared to control subjects

Participants with obesity had a significantly higher NSP (*P* < 0.0001) and lower sural (*P* < 0.0001) and peroneal (*P* = 0.006) nerve amplitudes, but no difference in sural and peroneal nerve conduction velocity and latency compared to controls (Table 1**)**. VPT (*P* < 0.0001) and WPT (*P* < 0.0001) were significantly higher, whilst CPT (*P* = 0.003) and HRV-DB (*P* < 0.0001) were significantly lower in participants with obesity compared to controls. CNFD (*P* < 0.0001), CNBD (*P* < 0.0001) and CNFL (*P* < 0.0001) were significantly lower in obese participants compared to controls.

### Obese participants with and without small nerve fibre damage

Based on a CNFL cut-off greater than 2 standard deviations below the mean of healthy controls, 51% of participants with obesity had significant small nerve fibre damage. More participants with small nerve fibre damage (58%) had metabolic syndrome compared to those without small fibre damage (23%; *P* = 0.02). CNFD (*P* < 0.0001), CNBD (*P* < 0.0001) and CNFL (*P* < 0.0001) were significantly lower in obese patients with small nerve fibre damage compared to those without. Other measures of neuropathy did not differ significantly between obese subjects with and without small nerve fibre damage, except for a higher sural nerve conduction velocity (*P* = 0.03) (Table 2).

**Table 2:** Clinical, metabolic and neuropathy measures in obese participants without (−ve) and with (+ ve) small nerve fibre damage.

Parameters	Obese (−ve) (n = 23)	Obese (+ ve) (n = 24)	*P*
*Demographics*			
Age (years)	47.4 ± 9.6	45.9 ± 9	0.9
Sex(Female/Male)	14/7	16/8	0.7
Ethnicity (Caucasian/Asian)	21/2	22/2	0.1
Smoking (no. per day)	0	2.15 ± 5.8	0.5
Alcohol (units per week)	2.3 ± 4.6	0.5 ± 1.1	0.7
Systolic BP (mmHg)	133.6 ± 22.1	126.7 ± 17.5	0.7
Diastolic BP (mmHg)	73.5 ± 11.0	71.9 ± 10.3	0.6
BMI (kg/m^2^)	50.6 ± 8.7	48.2 ± 8.2	0.1
Number on statin therapy (%)	3^13^	8^33^	0.1
Number (%) metabolic syndrome	5^23^	14^58^	0.02
HbA1c (mmol/mol)	38.5 ± 5.9	38.0 ± 4.6	0.9
Serum triglycerides (mmol/l)	1.4 ± 0.7	1.9 ± 1.3	0.02
LDL-C (mmol/l)	2.9 ± 0.8	2.8 ± 0.8	0.7
*Neuropathy Assessments*			
Neuropathy Symptom Profile	2.9 ± 4.0	4.7 ± 5.3	0.1
Neuropathy Disability Score	1.0 ± 1.7	1.3 ± 2.1	0.5
Vibration Perception Threshold (volts)	9.6 ± 5.6	11.7 ± 8.4	0.8
Sural Amplitude (μV)	10.33 ± 6.1	12.6 ± 10.7	0.3
Sural Velocity (m/s)	47.3 ± 1.9	51.3 ± 10.9	0.03
Peroneal amplitude (mV)	4.7 ± 2.6	3.4 ± 1.5	0.3
Peroneal velocity (m/s)	47.4 ± 6.3	46.3 ± 4.5	0.8
Cold Perception Threshold (°C)	25.9 ± 4.4	24.9 ± 6.2	0.5
Warm Perception Threshold (°C)	40.6 ± 3.2	40.7 ± 3.6	0.9
HRV-DB (beats per min)	20.0 ± 11.0	19.0 ± 12.0	0.8
CNFD (no/mm^2^)	29.8 ± 3.7	23.91 ± 3.9	< 0.0001
CNBD (no/mm^2^)	73.5 ± 21.7	42.2 ± 18.6	< 0.0001
CNFL (mm/mm^2^)	21.6 ± 2.5	15.4 ± 2.2	< 0.0001

Results reported as mean ± standard deviation; BMI (body mass index); BP ( blood pressure); eGFR (estimated glomerular filtration rate); HDL-C (high density lipoprotein cholesterol); LDL-C (low density lipoprotein cholesterol); HRV-DB (Heart rate variability-deep breathing; CNFD (corneal nerve fibre density); CNBD (corneal nerve branch density); CNFL (corneal nerve fibre length).

### Lipoproteins and HDL functionality markers

**Figure 1:**
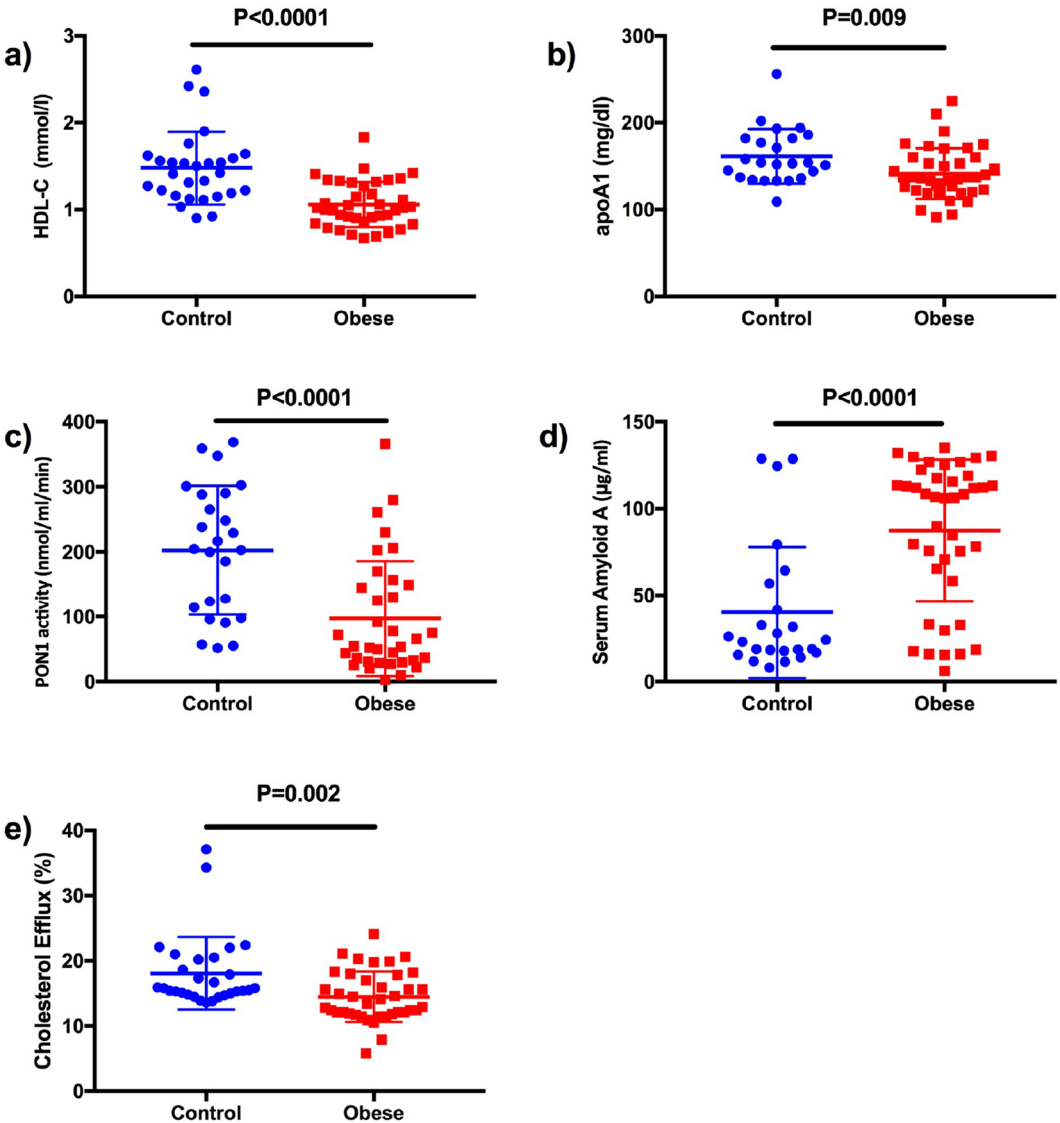
HDL cholesterol (HDL-C) and functionality between obese and control participants. (**a**) HDL cholesterol, (**b**) Apolipoprotein A1, (**c**) Paraxonase 1 activity, (**d**) Serum Amyloid A, (**e**) HDL’s capacity to promote cholesterol efflux in vitro.

Eleven (23%) of the participants with severe obesity were treated with statins, but their lipid profile, apoA1, apoB and HDL functionality markers did not differ from participants not on statins. Total cholesterol was significantly lower (*P* = 0.002), but there was no significant difference in LDL-C or serum triglycerides in participants with severe obesity compared to controls. HDL-C (*P* < 0.0001), PON-1 (*P* < 0.0001), apoA1 (*P* = 0.009) and cholesterol efflux (*P* = 0.002) were lower, and SAA (*P* < 0.0001) was higher in obese participants compared to controls **(**Fig. 1**)**. Serum triglycerides (*P* = 0.02) were higher and PON-1 activity (*P* = 0.005) was lower in obese patients with small nerve fibre damage; however, HDL-C (*P* = 0.8), cholesterol efflux (*P* = 0.08), apoA1 (*P* = 0.8) and SAA (*P* = 0.8) did not differ between obese participants with and without small nerve fibre damage (Table 3).

**Table 3:** HDL functionality in control and obese participants with and without small fibre neuropathy.

Parameters	Control (n = 30)	Obese (-ve) (n = 23)	Obese (+ ve) (n = 24)	*P* value
HDL-C (mmol/l)	1.5 ± 0.42	1.1 ± 0.27*	1.1 ± 0.26*	< 0.0001
apoA1 (mg/dl)	161.2 ± 31.32	142.1 ± 39.32^#^	140.7 ± 21.5^#^	< 0.0001
PON-1 activity (nmol/ml/min)	202.7 ± 99.27	141.8 ± 107.4*	50.8 ± 47.03^$^*	0.02
SAA (μg/ml)	40.1 ± 37.93	89.9 ± 38.5*	83.6 ± 48.49*	0.002
Cholesterol efflux (%)	18.1 ± 5.58	13.5 ± 3.64*	15.8 ± 4.01	0.002

Results reported as mean ± standard deviation. HDL-C, high density lipoprotein cholesterol; apoA1, apolipoprotein A1; PON-1, paraoxonase-1; SAA, serum amyloid A.

*indicates *P* < 0.01 compared to control.

^#^indicates *P* < 0.05 compared to controls.

^$^indicates *P* < 0.01 compared to obese without small fibre damage.

## Discussion

We have shown significant small nerve fibre damage in participants with severe obesity which was associated with reduced PON-1 activity, higher serum triglycerides and metabolic syndrome. Experimental studies have shown that high-fat fed mice develop neuropathy^22^. We and others have previously demonstrated significant small fibre damage in people with metabolic syndrome and IGT^3,5,12^. A study has shown that 11% of women with severe obesity have peripheral neuropathy^32^ and we have recently reported small nerve fibre regeneration after bariatric surgery^33^ .

The exact mechanisms underlying small nerve fibre damage in obesity are not understood; but appear to be different to the hyperglycemia-mediated large fibre neuropathy in patients with diabetes^34^. Patients with severe obesity have small fibre dysfunction^8^ and a reduction in tibial and peroneal nerve amplitudes^9^, which correlates with BMI^35^. Participants with moderate obesity showed reduced sensory nerve amplitudes and normal nerve conduction velocity which was attributed to impaired percutaneous stimulation due to thicker subcutaneous tissue^36^. In the present study we show that individuals with obesity without diabetes have evidence of small nerve fibre damage, evidenced by corneal nerve loss. These observations are consistent with the Utah Diabetic Neuropathy Study which reported that obesity and hypertriglyceridemia were related to a loss of intraepidermal nerve fibres^12^. In the present study we also identify cardiac autonomic dysfunction in individuals with obesity. Central obesity has been associated with cardiac autonomic neuropathy in participants with impaired glucose tolerance^37^, and Valensi et al. have previously shown that deep breathing heart rate variability correlates with BMI in type 2 diabetes^10^. Furthermore, in a series of 121 participants with obesity, 50% had at least one abnormal cardiac autonomic function test^38^. In individuals with obesity, waist-to-hip ratio was associated with reduced parasympathetic and increased sympathetic activation^39^ and obesity predicts the development of autonomic neuropathy in type 2 diabetes^40^.

The EURODIAB study reported a significant association between total cholesterol and serum triglyceride levels with incident diabetic peripheral and cardiac autonomic neuropathy in patients with type 1 diabetes^18^. Wiggin et al.^4^ showed that elevated serum triglycerides predicted a loss of myelinated nerve fibres in sural nerve biopsies from people with diabetes^41^; and the DISTANCE study showed that serum triglycerides were an independent risk factor for non-traumatic lower extremity amputation^42^. Indeed, in the present study we report a significantly higher level of serum triglycerides and prevalence of metabolic syndrome in people with obesity and small nerve fibre damage.

HDL is the most abundant lipoprotein in human tissue and provides protection to cell membranes from oxidative stress^43^. Intracellular and membrane cholesterol distribution are important for neuronal integrity, but excess cholesterol is detrimental and can promote amyloid precursor protein cleavage and the generation of toxic amyloid peptides^44^. Maintaining a physiologic cholesterol balance is vital for neuronal function and synaptic transmission^45^. Cholesterol efflux and the capacity of HDL to accept cholesterol is partly regulated by ATP-binding cassette subfamily A1 (ABCA1) and ATP-binding cassette subfamily G1 (ABCG1) proteins^46^. Tangier disease is a rare inherited metabolic neuropathy due to a mutation in the ABCA1 gene which results in a marked reduction in circulating HDL particles and serum HDL-C level and accumulation of cholesterol esters in peripheral nerves^47^. HDL is not only involved in reverse cholesterol transport, but also impedes lipid peroxidation and has anti-inflammatory properties^43^. ApoA1, the main protein component in HDL, is known to have an anti-inflammatory effect and inhibit dendritic cell differentiation^48^. We demonstrate significantly reduced PON-1 activity in people with obesity and small nerve fibre damage. PON-1 is an HDL associated enzyme with anti-oxidant/glycation properties, and certain PON-1 genotypes increase the risk of developing microalbuminuria and retinopathy^49^. PON-1 activity also correlates with the capacity of HDL to protect LDL against oxidation in vitro^19^, hence decreased PON-1 activity may lead to neuropathy through a mechanism involving increased lipid peroxidation. Low PON-1 activity has been implicated in the development of retinopathy^50^ and macrovascular disease^51^ in patients with diabetes; and Abbott et al.^52^ demonstrated lower PON-1 activity in patients with diabetic neuropathy. Fenofibrate increases PON-1 activity^53^, which may account for its benefit in reducing amputations^54^.

SAA is an acute phase protein, which correlates with BMI and decreases with weight loss^55^. There is increasing evidence for its role in adipose tissue inflammation, insulin resistance, diabetes and cardiovascular disease^56^. During an acute phase reaction, SAA displaces apoA1, compromising HDL’s capacity to protect against oxidation and promote cholesterol efflux^56^. Recent studies have shown that the association between obesity and incident diabetic neuropathy may be partially mediated by inflammation^7^. We show that patients with obesity have a significantly higher SAA and lower capacity of HDL to promote cholesterol efflux; however, this did not differ between subjects with and without small nerve fibre damage. We also report abnormalities in multiple markers of HDL functionality in people with obesity perse which have been reported in patients with cardiovascular disease^57^ and patients with rheumatoid arthritis^58^, suggesting a common pathway for inflammation and enhanced vascular disease risk in these patients.

In conclusion we show there is evidence of small nerve fibre damage in people with severe obesity. Patients with obesity had elevated serum triglycerides and SAA and lower HDL-C, PON-1 activity and cholesterol efflux. Furthermore, obese subjects with small nerve fibre damage, compared to those without, had higher serum triglycerides and prevalence of metabolic syndrome and lower PON1 activity. These factors may represent therapeutic targets to prevent or reverse small nerve fibre damage in obesity.

## Data Availability

The data is available from the corresponding author with reasonable request.

## Abbreviations

apoA1: Apolipoprotein AI
apoB: Apolipoprotein B
ABCA1 and ABCG1: ATP-binding cassette subfamily A
CV: Coefficient of variation
CPT: Cold perception threshold
CNBD: Corneal nerve branch density
CNFD: Corneal nerve fiber density
CNFL: Corneal nerve fiber length
HRV-DB: Heart rate variability with deep breathing
IGT: Impaired glucose tolerance
IL: Interleukin
IENFD: Intra-epidermal nerve fibre density
LOX1: Lectin-like oxLDL receptor-1
NDS: Neuropathy disability score
MPO: Myeloperoxidase
NSP: Neuropathy symptom profile
NADPH: Nicotinamide adenine dinucleotide phosphate
oxLDL: Oxidised LDL
PON-1: Paraoxonase-1
PN: Peripheral neuropathy
SFN: Small fibre neuropathy
SAA: Serum Amyloid A
VPT: Vibration perception threshold
WPT: Warm perception threshold
